# Supplementary material for: Purifying selection shapes the dynamics of P-element invasion in Drosophila simulans populations
Source: Genome Biol. 2025 Jul 24;26:221. doi: 10.1186/s13059-025-03688-2 (PMC12291496; doi:10.1186/s13059-025-03688-2)
Supplement: Supplementary file 1 — Additional file 1. [file 13059_2025_3688_MOESM1_ESM.pdf]

# Additional File 1: Supplementary Information

## Supplementary Tables

**Table S1.** Model parameters of the extended individual-based P-element invasion model and their considered ranges, including additional model parameters for excision probability (*v*) and dominance (*h*). The range for $p_{carrier}$ was guided by a previous study estimating that 25 – 44% of the isofemale lines used in the 1st and 2nd wave experiment carried the P-element (34). The range for $f_{regulatory}$ was based on the estimate that 3.5% of the *Drosophila melanogaster* genome consists of piRNA clusters with TE-regulatory properties (38). However, because piRNA clusters are challenging to assemble and compare across species (54) and because a previous simulation study suggests that as little as 0.2% may be sufficient for TE control (55), we allowed $f_{regulatory}$ to vary rather than fixing it. The range for *u* was informed by the observed P-element invasion dynamics in the 1st wave experiment. Specifically, the lower bound of 0.15 was chosen based on effective transposition rate estimates derived from generations 0 and 10 in the 1st wave experiment. The upper bound of 0.5 ensures that effective transposition remains possible even under strong purifying selection scenarios.

| **Parameter** | **Description** | **Range** |
|---|---|---|
| $p_{carrier}$ | The probability that an isofemale line carries the P-element at the beginning of the simulation | [0.15, 0.5] |
| $f_{regulatory}$ | piRNA cluster size: the fraction of the chromosome with P-element-regulatory properties (once at least one P-element insertion is acquired) | [0.01, 0.1] |
| *u* | The probability of transposition of a single P-element per generation | [0.15, 0.5] |
| *v* | The probability of a clean excision event at the original insertion site, given that a P-element transposes | [0, 0.25] |
| $\alpha$ | The shape parameter $\alpha$ of the beta distribution that defines the selection coefficient *s* for P-element insertions outside piRNA clusters | [0.001, 0.5] |
| $\beta$ | The shape parameter $\beta$ of the beta distribution that defines the selection coefficient *s* for P-element insertions outside piRNA clusters | [10, 20] |
| *h* | The dominance coefficient specific to each P-element insertion | [0, 1] |

**Table S2.** Summary of top-ranked parameter combinations from the parameter exploration with the Gaussian Process model trained on the extended individual-based P-element invasion model that includes additional parameters for excision probability ($v$) and dominance ($h$). Parameter combinations are grouped *post hoc* by mean selection efficacy ( $2 \times h \times |\bar{s}| \times N_e$) under the assumption of $N_e$ = 221 (59). Brackets indicate the range across the top 100 parameter combinations ranked by lowest $NRMSE_{sum}$. Variation among the top 100 parameter combinations reflects that agreement between GP predictions and experimental data depends on specific combinations of model parameters, and that the fitness effects of new P-element insertions arise from the product of $h \times s$, limiting the interpretability of $s$ or $h$ individually.

| **Parameter/ Metric** | **Description** | **Mean selection efficacy** $2 \times h \times \lvert\bar{s}\rvert \times N_e > 1$ | $2 \times h \times \lvert\bar{s}\rvert \times N_e \leq 1$ |
|---|---|---|---|
| $p_{carrier}$ | The probability that an isofemale line carries the P-element at the beginning of the simulation | 0.215 [0.153; 0.260] | 0.163 [0.150; 0.228] |
| $f_{regulatory}$ | The piRNA cluster size | 0.014 [0.010; 0.022] | 0.083 [0.074; 0.092] |
| $u$ | Transition probability of a single P-element per generation | 0.339 [0.268; 0.391] | 0.347 [0.316; 0.428] |
| $v$ | Excision probability at the original insertion site given that a P-element transposes | 0.188 [0.004; 0.244] | 0.036 [0.000; 0.182] |
| $\alpha$ | The $\alpha$ parameter of the beta distribution that determines the selection coefficient $s$ for P-elements outside piRNA clusters | 0.323 [0.284, 0.499] | 0.385 [0.107; 0.498] |
| $\beta$ | The $\beta$ parameter of the beta distribution that determines $s$ for P-elements outside piRNA clusters | 11.766 [10.072; 19.976] | 10.785 [10.135; 19.513] |
| $h$ | Dominance coefficient | 0.749 [0.488; 0.967] | 0.066 [0.032; 0.362] |
| $\lvert\bar{s}\rvert$ | Mean selection coefficient for new P-element insertions outside piRNA clusters; $(\mathrm{E}(\lvert s\rvert) = \lvert\bar{s}\rvert = \alpha/(\alpha+\beta))$ | 0.027 [0.018; 0.044] | 0.034 [0.006; 0.042] |
| $95^{th}$ percentile $\lvert s\rvert$ | $95^{th}$ percentile of $\lvert s\rvert$ for new P-element insertions outside piRNA clusters; | 0.119 [0.079; 0.165] | 0.143 [0.035; 0.163] |
| Neutral fraction | Expected fraction of effectively neutral $(2 \times h \times \lvert s\rvert \times N_e \leq 1)$ P-element insertions outside of piRNA clusters | 0.374 [0.233; 0.407] | 0.696 [0.684; 0.822] |
| $\lvert\bar{s}_{\text{effective}}\rvert$ | Mean selection coefficient for new P-element insertions outside piRNA clusters where selection is strong enough to overcome genetic drift $(2 \times h \times \lvert s\rvert \times N_e > 1)$ | 0.042 [0.029; 0.058] | 0.094 [0.031; 0.133] |
| $NRMSE_{sum}$ | Fit metric: $NRMSE_{sum}$ = NRMSE $1^{st}$ + NRMSE $2^{nd}$ wave NRMSE = normalized root mean square error | 1.353 [1.353; 1.418] | 2.196 [2.196; 2.266] |
| Best $NRMSE_{sum}$ Rank | Rank of the parameter combination with the minimum $NRMSE_{sum}$ within group | 1 [1; 100] | 123217 [123217; 176086] |

**Table S3.** Summary of top-ranked parameter combinations from the parameter exploration with the Gaussian Process model. Parameter combinations are grouped *post hoc* by mean selection efficacy ( $|\bar{s}| \times N_e$) under the assumption of $N_e$ = 442 and co-dominance. Brackets indicate the range across the top 100 parameter combinations ranked by lowest $NRMSE_{sum}$.

| **Parameter/ Metric** | **Description** | **Mean selection efficacy** $|\bar{s}| \times N_e > 1$ | $|\bar{s}| \times N_e \leq 1$ |
|---|---|---|---|
| $p_{carrier}$ | The probability that an isofemale line carries the P-element at the beginning of the simulation | 0.204 [0.155; 0.257] | 0.151 [0.151; 0.497] |
| $f_{regulatory}$ | The piRNA cluster size | 0.017 [0.013; 0.028] | 0.098 [0.092; 0.100] |
| *u* | Transition probability of a single P-element per generation | 0.290 [0.246; 0.324] | 0.343 [0.308; 0.367] |
| $\alpha$ | The $\alpha$ parameter of the beta distribution that determines the selection coefficient *s* for P-elements outside piRNA clusters | 0.461 [0.339; 0.500] | 0.039 [0.009; 0.043]- |
| $\beta$ | The $\beta$ parameter of the beta distribution that determines *s* for P-elements outside piRNA clusters | 10.743 [10.128; 15.860] | 17.379 [10.026; 19.862] |
| $|\bar{s}|$ | Mean selection coefficient for new P-element insertions outside piRNA clusters; $(\mathrm{E}(|s|) = |\bar{s}| = \alpha/(\alpha+\beta))$ | 0.041 [0.030; 0.046] | 0.002 [0.001; 0.002] |
| 95th percentile $|s|$ | 95th percentile of $|s|$ for new P-element insertions outside piRNA clusters; | 0.160 [0.115; 0.173] | 0.011 [0.000; 0.011] |
| Neutral fraction | Expected fraction of effectively neutral ($|s| \times N_e \leq 1$) P-element insertions outside of piRNA clusters | 0.199 [0.170; 0.310] | 0.898 [0.892; 0.971] |
| $|\bar{s}_{\mathrm{effective}}|$ | Mean selection coefficient for new P-element insertions outside piRNA clusters where selection is strong enough to overcome genetic drift ($|s| \times N_e > 1$) | 0.051 [0.038; 0.055] | 0.021 [0.019; 0.031] |
| $NRMSE_{sum}$ | Fit metric: $NRMSE_{sum}$ = NRMSE 1st + NRMSE 2nd wave<br>NRMSE = normalized root mean square error | 1.340 [1.340; 1.509] | 2.313 [2.313; 2.388] |
| Best $NRMSE_{sum}$ Rank | Rank of the parameter combination with the minimum $NRMSE_{sum}$ within group | 1 [1; 100] | 38394 [38394; 56323] |

**Table S4.** Summary of top-ranked parameter combinations from the parameter exploration with the Gaussian Process model trained on the extended individual-based P-element invasion model that includes additional parameters for excision probability (*v*) and dominance (*h*). Parameter combinations are grouped *post hoc* by mean selection efficacy ($2 \times h \times |\bar{s}| \times N_e$) under the assumption of $N_e$ = 442. Brackets indicate the range across the top 100 parameter combinations ranked by lowest $NRMSE_{sum}$. Variation among the top 100 parameter combinations reflects that agreement between GP predictions and experimental data depends on specific combinations of model parameters, and that the fitness effects of new P-element insertions arise from the product of $h \times s$, limiting the interpretability of *s* or *h* individually.

| Parameter/ Metric | Description | Mean selection efficacy $2 \times h \times \lvert\bar{s}\rvert \times N_e > 1$ | Mean selection efficacy $2 \times h \times \lvert\bar{s}\rvert \times N_e \le 1$ |
|---|---|---|---|
| $p_{carrier}$ | The probability that an isofemale line carries the P-element at the beginning of the simulation | 0.215 [0.153; 0.260] | 0.172 [0.150; 0.335] |
| $f_{regulatory}$ | The piRNA cluster size | 0.014 [0.010; 0.022] | 0.087 [0.076; 0.100] |
| *u* | Transition probability of a single P-element per generation | 0.339 [0.268; 0.391] | 0.352 [0.308; 0.500] |
| *v* | Excision probability at the original insertion site given that a P-element transposes | 0.188 [0.004; 0.244] | 0.071 [0.002; 0.241] |
| $\alpha$ | The $\alpha$ parameter of the beta distribution that determines the selection coefficient *s* for P-elements outside piRNA clusters | 0.323 [0.284, 0.499] | 0.429 [0.002; 0.499] |
| $\beta$ | The $\beta$ parameter of the beta distribution that determines *s* for P-elements outside piRNA clusters | 11.766 [10.072; 19.976] | 11.058 [10.023; 19.285] |
| *h* | Dominance coefficient | 0.749 [0.488; 0.967] | 0.019 [0.002; 0.978] |
| $\lvert\bar{s}\rvert$ | Mean selection coefficient for new P-element insertions outside piRNA clusters; ($\mathrm{E}(\lvert s\rvert) = \lvert\bar{s}\rvert = \alpha/(\alpha+\beta)$) | 0.027 [0.018; 0.044] | 0.037 [0.000; 0.045] |
| 95th percentile $\lvert s\rvert$ | 95th percentile of $\lvert s\rvert$ for new P-element insertions outside piRNA clusters; | 0.119 [0.079; 0.165] | 0.149 [0.000; 0.171] |
| Neutral fraction | Expected fraction of effectively neutral ($2 \times h \times \lvert s\rvert \times N_e \le 1$) P-element insertions outside of piRNA clusters | 0.300 [0.168; 0.334] | 0.786 [0.681; 1.000] |
| $\lvert\bar{s}_{\mathrm{effective}}\rvert$ | Mean selection coefficient for new P-element insertions outside piRNA clusters where selection is strong enough to overcome genetic drift ($2 \times h \times \lvert s\rvert \times N_e > 1$) | 0.038 [0.026; 0.053] | 0.120 [0.019; 0.509] |
| $NRMSE_{sum}$ | Fit metric: $NRMSE_{sum}$ = NRMSE 1st + NRMSE 2nd wave<br>NRMSE = normalized root mean square error | 1.353 [1.353; 1.418] | 2.295 [2.295; 2.338] |
| Best $NRMSE_{sum}$ Rank | Rank of the parameter combination with the minimum $NRMSE_{sum}$ within group | 1 [1; 100] | 205378 [205378; 258411] |

## Supplementary Figures

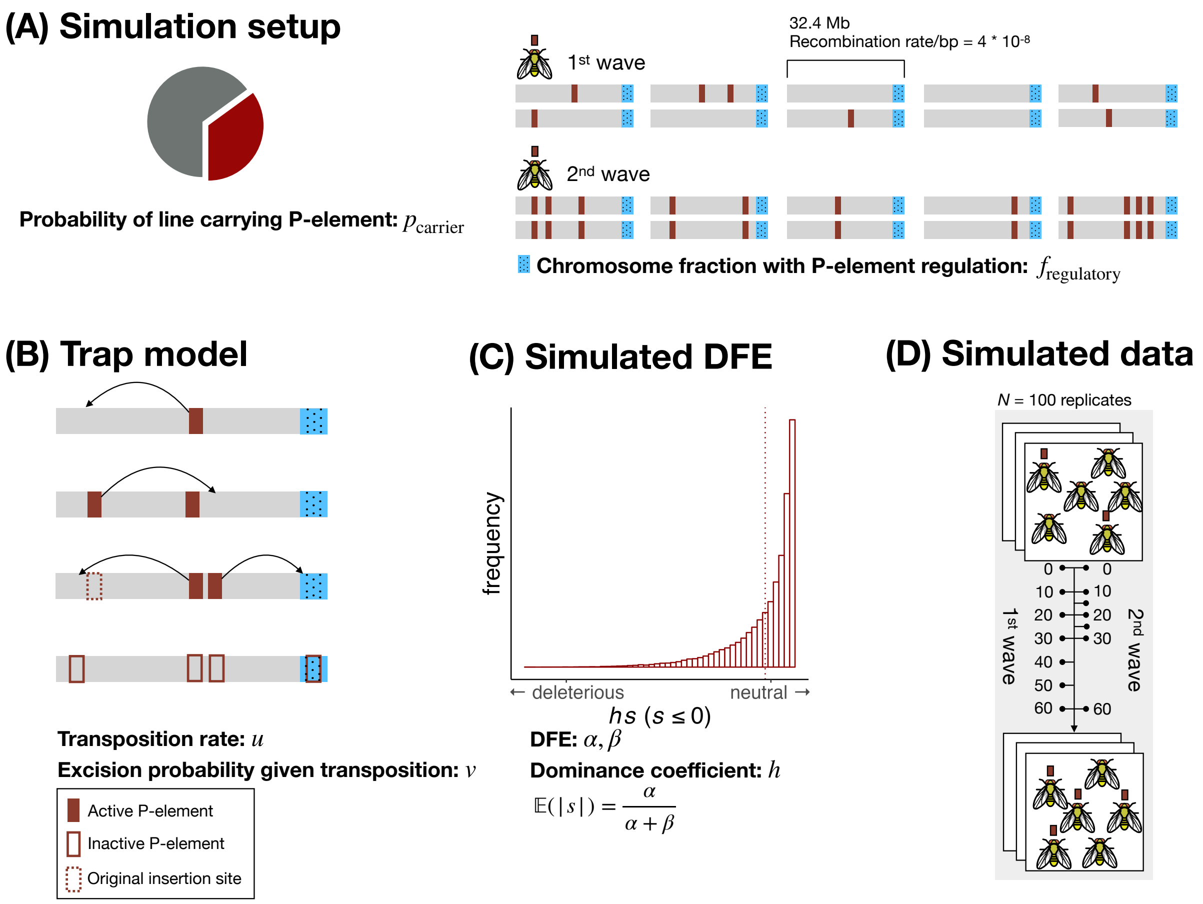


**Figure S1.** Schematic overview of the extended individual-based simulation model mimicking the P-element invasion dynamics in our experimental evolution experiments, including additional model parameters for excision probability ($v$) and dominance ($h$). **(A) Simulation setup:** Ancestral outbred populations are generated by mixing 200 isofemale lines (five flies each). Each line carries the P-element with probability $p_{carrier}$. We modeled diploid individuals with five chromosomes, each with a fixed length of 32.4 Mb and a recombination rate of $4{\times}10^{-8}$ per bp per generation. For the 1st wave, P-element insertions are assumed to be heterozygous. For the 2nd wave, since the experiment started with isofemale lines that had been maintained at small populations sizes for 4.5 years (11), the model assumes that all P-element insertions are homozygous due to increased inbreeding and the likely establishment of a defense mechanism. The parameter $f_{regulatory}$ defines the fraction of each chromosome with P-element-regulatory properties (piRNA clusters; blue rectangles). **(B) Trap model:** The P-element remains active (filled rectangle) unless one of its copies transposes into a piRNA cluster (blue rectangles). The probability that a single P-element undergoes transposition in a given generation is controlled by the transposition rate $u$. If transposition occurs, the probability of a clean excision event at the original insertion site — represented by an unfilled rectangle with dotted red borders — is determined by the parameter $v$. Once a piRNA cluster acquires a single P-element insertion, all P-elements in the genome are immediately inactivated (unfilled rectangles with solid red borders). **(C) Simulated distribution of fitness effects (DFE):** The DFE for new P-element insertions is modeled using a beta distribution with shape parameters $\alpha$ and $\beta$, which define the selection coefficient $s$. Each new P-element insertion is also assigned a dominance coefficient $h$ ranging between 0 and 1. Positive selection is not considered in our model. **(D) Simulated data:** The simulation output used in the analyses contains the average P-element copy number per haploid genome across 100 simulation runs, taken at the same time points used in the two experimental evolution studies.

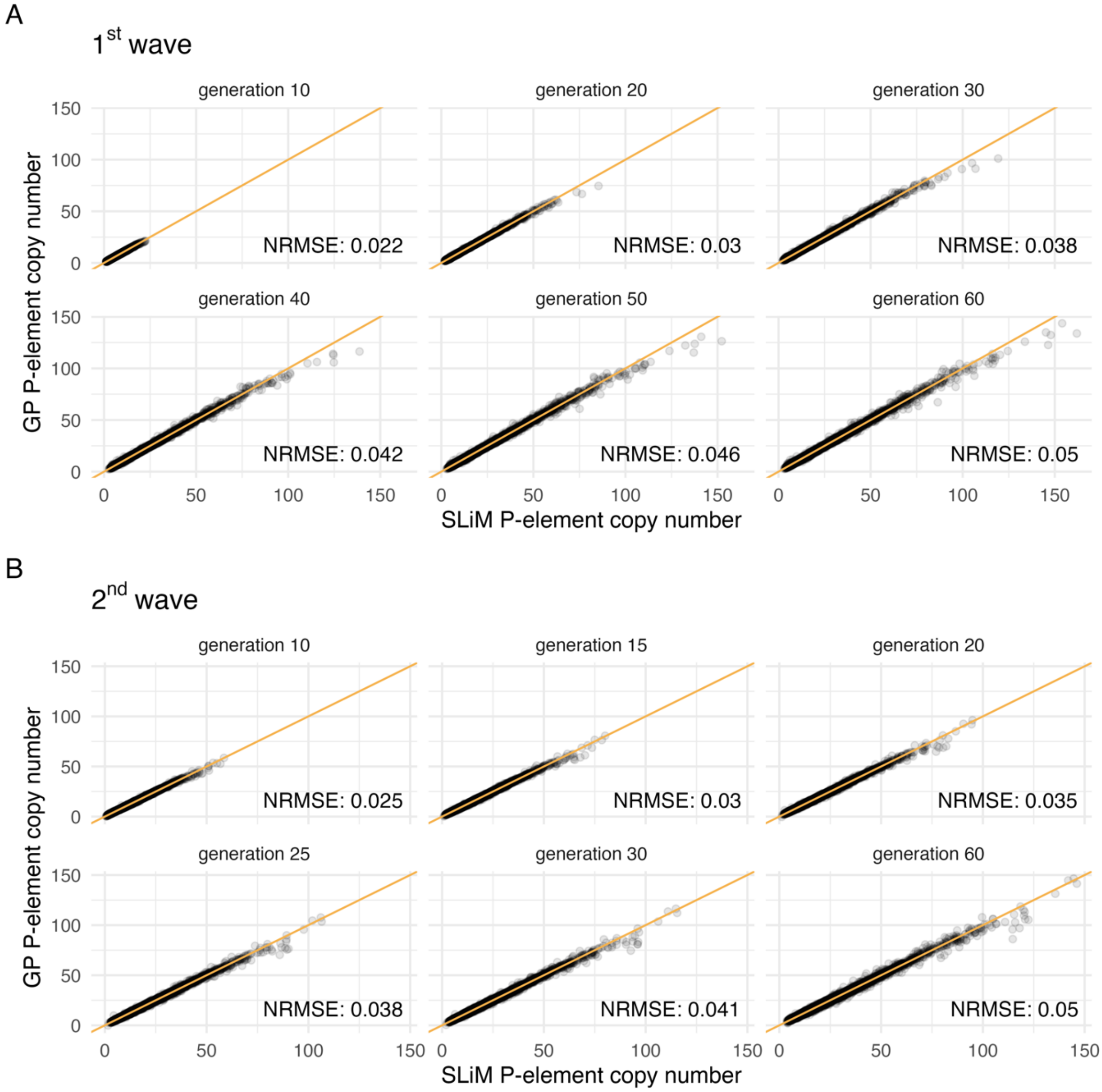


**Figure S2.** Gaussian Process (GP) performance: A test dataset consisting of 10000 data points was simulated with the extended individual-based model (*x* axis) for the (A) 1$^{st}$ wave and (B) 2$^{nd}$ wave and compared to the predictions of the GP (*y* axis). One data point in this test dataset comprises a specific combination of seven model parameters (Additional file 1: Table S1) and the corresponding predicted P-element copy numbers for six time points. The observed and predicted P-element copy numbers are shown (gray dots) for each of those six time points (the six panels), with amber lines indicating the identity line ($x = y$). Normalized root mean square errors (NRMSE) for the time points are shown at the bottom right of the corresponding panels. The analysis shows that the GP can predict the copy number observed in the individual-based model very accurately. Only at extremely high copy numbers — well beyond the empirical estimates (Figure 2) — can a slight underestimation be observed.

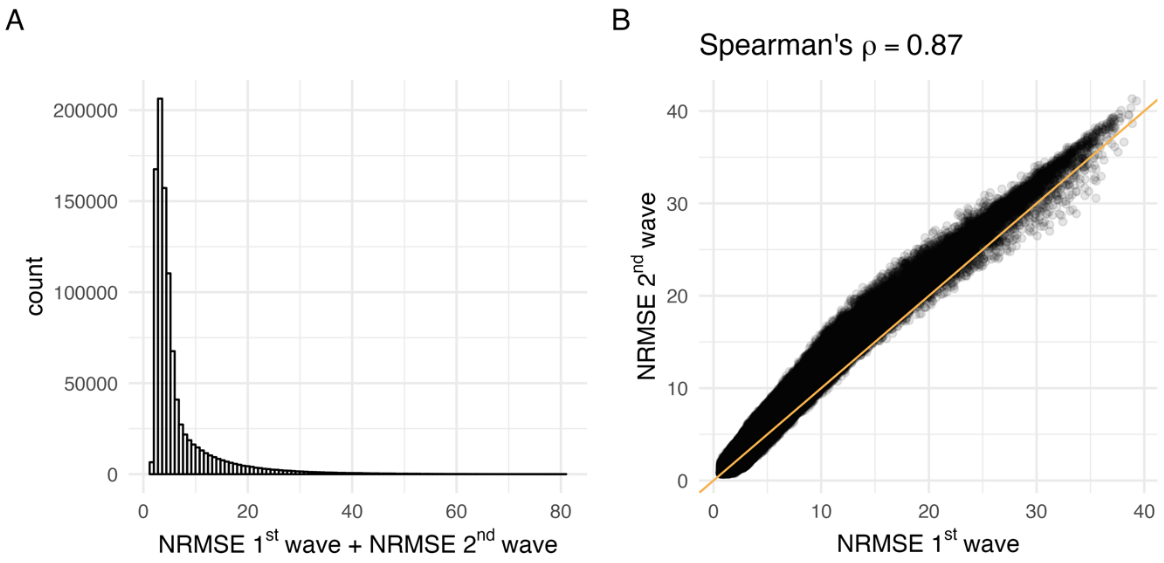


**Figure S3.** Normalized root mean square error (NRMSE) for GP predictions using $10^6$ different parameter combinations. (A) Distribution of NRMSE sums, showing variation in the agreement between GP predictions and empirical data. While most values are moderate, a few combinations have very low or very high NRMSE values. (B) Comparison of NRMSE for the 1st wave (*x* axis) versus the 2nd wave (*y* axis). Each point represents the NRMSEs for one of the $10^6$ data points. The amber line represents the identity line ($x = y$). Overall, there is strong agreement between the NRMSEs, with a Spearman rank correlation coefficient of 0.87.

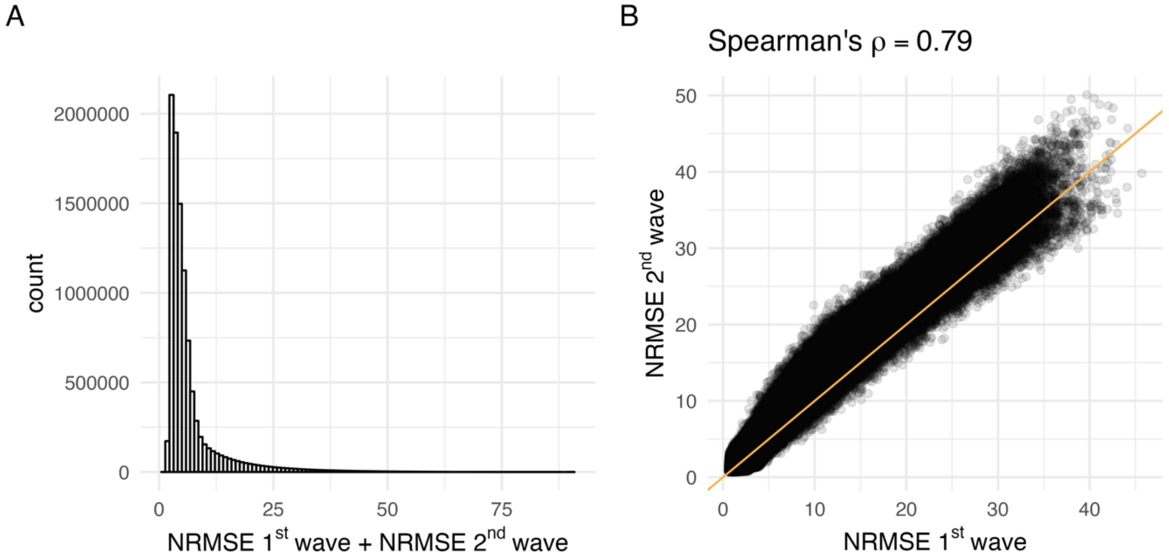


**Figure S4.** Normalized root mean square error (NRMSE) for predictions made by GPs trained on simulation outcomes from the extended individual-based model. (A) Distribution of NRMSE sums, showing variation in the agreement between GP predictions using $10^7$ different parameter combinations and empirical data. While most values are moderate, a few combinations have very low or very high NRMSE values. (B) Comparison of NRMSE for the 1st wave (*x* axis) versus the 2nd wave (*y* axis). Each point represents the NRMSEs for one of the $10^7$ data points. The amber line represents the identity line ($x = y$). Overall, there is strong agreement between the NRMSEs, with a Spearman rank correlation coefficient of 0.79.

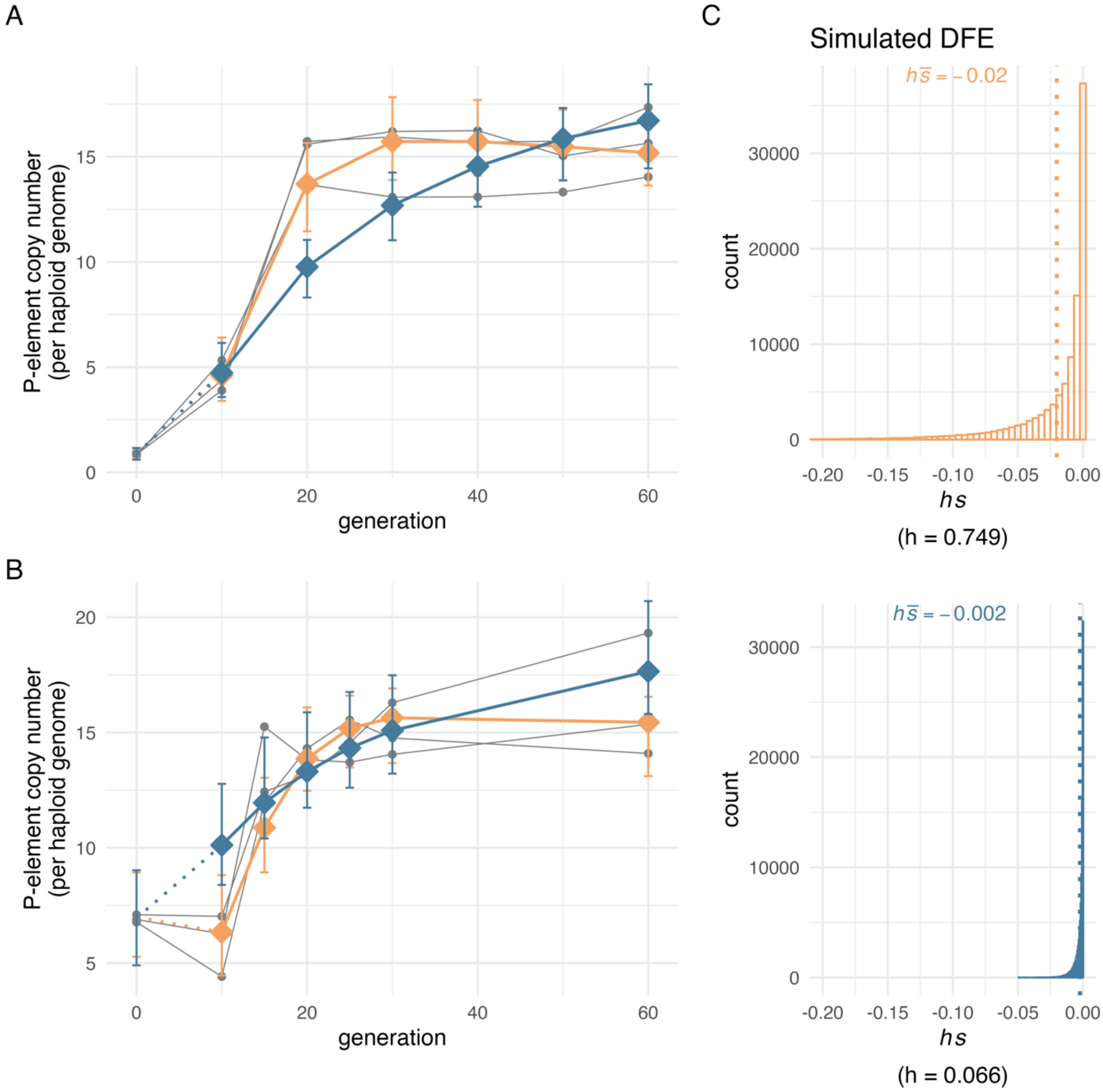


**Figure S5.** Gaussian Process (GP) predictions provide a good fit to the empirical data — the top-ranked parameters were identified using 1st and 2nd wave data. GPs were trained on simulation outcomes of the extended individual-based model. GP predictions from the parameter combinations with the lowest $NRMSE_{sum}$ when compared against empirical data are shown for scenarios with expected effective purifying selection ( $2 \times h \times |\bar{s}| \times N_e > 1$ , amber), and for scenarios without expected effective purifying selection ( $2 \times h \times |\bar{s}| \times N_e \leq 1$, steel blue) assuming $N_e$ = 221 (59). Each grey line represents an empirical evolution replicate, with sequenced time points indicated by dots. GP predictions are indicated by connected colored diamonds. The error bars show the range between the 2.5th and 97.5th percentiles of P-element copy number trajectories simulated with the individual-based model. These simulations were run with the same parameters as those used for GP prediction. The error bars are shown at the same time points where empirical data were sequenced. Note that GP predictions do not cover generation 0; instead, predictions from generation 10 are connected to the individual-based simulation averages at generation 0 to aid visual interpretation. (A) 1st wave experiment. (B) 2nd wave experiment. (C) Simulated distribution of fitness effects (DFE) used in (A) and (B). Due to selection, the actual distribution of selection coefficients for segregating P-element insertions in the simulated populations will be skewed toward 0. Results are robust across the 100 parameter combinations with the lowest NRMSE sums (Additional file 1: Figure S7).

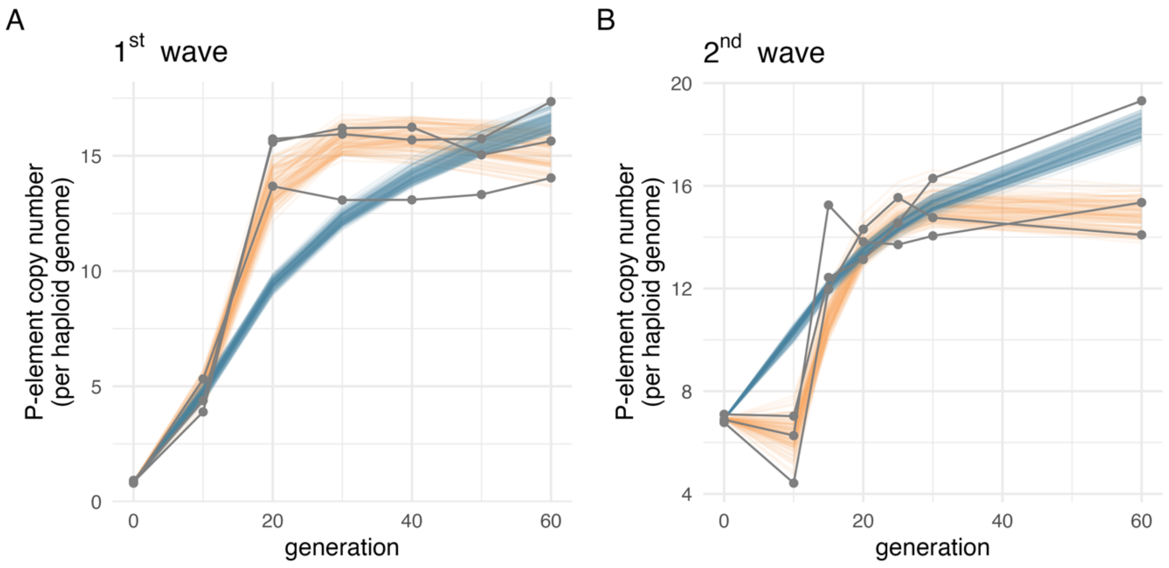


**Figure S6.** Individual Gaussian Process (GP) predictions for the top-ranked parameter combinations identified through parameter exploration. Predictions are grouped *post hoc* based on the mean selection efficacy: scenarios with expected effective purifying selection ($|\bar{s}| \times N_e > 1$, amber) and scenarios without ($|\bar{s}| \times N_e \leq 1$, steel blue), assuming $N_e$ = 221 (59). For each group, predictions from the 100 top-ranked parameter combinations (based on lowest $NRMSE_{sum}$, Table 2) are shown. Each grey line represents an empirical evolution replicate, with sequenced time points indicated by dots. Note that GP predictions do not cover generation 0; instead, predictions from generation 10 are connected to the empirical average at generation 0 to aid visual interpretation.

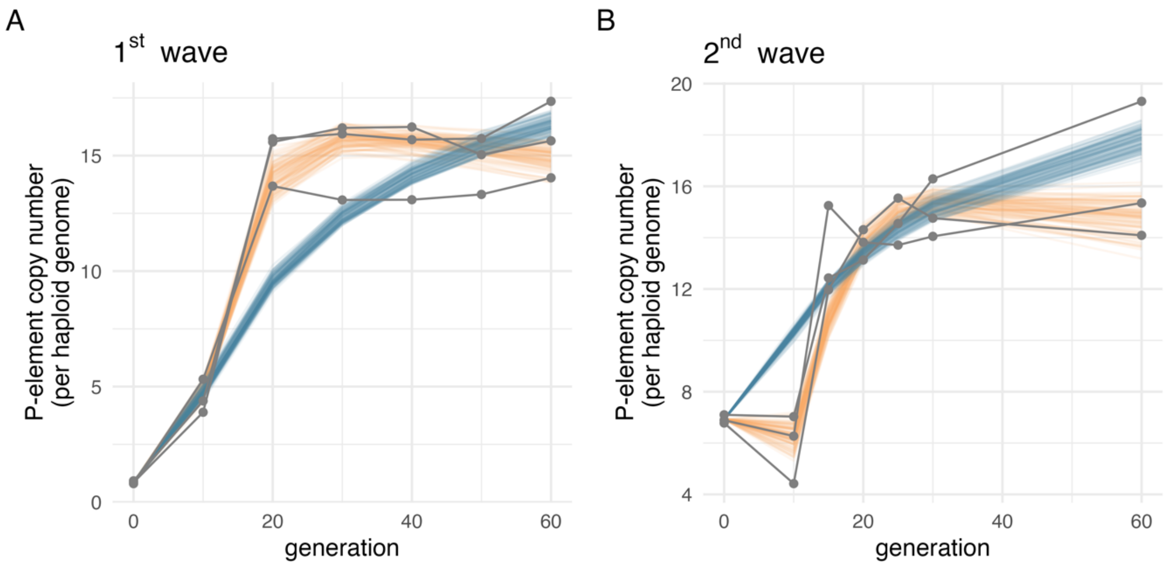


**Figure S7.** Individual Gaussian Process (GP) predictions for the top-ranked parameter combinations identified through parameter exploration. GPs were trained on simulation outcomes of the extended individual-based model. Predictions are grouped *post hoc* based on the mean selection efficacy: scenarios with expected effective purifying selection ($2 \times h \times |\bar{s}| \times N_e > 1$, amber) and scenarios without ($2 \times h \times |\bar{s}| \times N_e \leq 1$, steel blue), assuming $N_e$ = 221 (59). For each group, predictions from the 100 top-ranked parameter combinations (based on lowest $NRMSE_{sum}$, Additional file 1: Table S2) are shown. Each grey line represents an empirical evolution replicate, with sequenced time points indicated by dots. Note that GP predictions do not cover generation 0; instead, predictions from generation 10 are connected to the empirical average at generation 0 to aid visual interpretation.
